# Biomechanical analysis of two medial buttress plate fixation methods to treat Pauwels type III femoral neck fractures

**DOI:** 10.1186/s12891-022-05014-4

**Published:** 2022-01-14

**Authors:** Jichao Liu, Zhengwei Li, Jie Ding, Bingzhe Huang, Chengdong Piao

**Affiliations:** 1 Department of Orthopedics, The Second Hospital of Jilin University, 218 Ziqiang Road, Nanguan Street, Changchun, 130041 Jilin Province China; 2 Department of Stomatology, The Affiliated Hospital of Changchun University of Traditional Chinese Medicine, Changchun, Jilin China

**Keywords:** Femoral neck fracture, Biomechanics, Pauwels III, Medial buttress plate

## Abstract

**Background:**

Femoral neck fractures in young people are usually Pauwels Type III fractures. The common treatment method are multiple parallel cannulated screws or dynamic hip screw sliding compression fixation. Due to the huge shear stress, the rate of complications such as femoral head necrosis and nonunion is still high after treatment. The aim of our study was to compare the stabilities of two fixation methods in fixating pauwels type III femoral neck fractures.

**Methods:**

All biomimetic fracture samples are fixed with three cannulated screws combined with a medial buttress plate. There were two fixation groups for the buttress plate and proximal fracture fragment: Group A, long screw (40 mm); Group B, short screw (6 mm). Samples were subjected to electrical strain measurement under a load of 500 N, axial stiffness was measured, and then the samples were axially loaded until failure. More than 5 mm of displacement or synthetic bone fracture was considered as construct failure.

**Results:**

There were no significant differences in failure load (*P* = 0.669), stiffness (*P* = 0.842), or strain distribution (*P* > 0.05) between the two groups.

**Conclusions:**

Unicortical short screws can provide the same stability as long screws for Pauwels Type III Femoral Neck Fractures.

## Introduction

Femoral neck fractures are fairly common in clinical work, but they are rare in young patients (20–55 years old), who account for only about 3–5% of all cases [1, 2]. Femoral neck fractures in young people are typically high-energy vertical shear injuries, and most are Pauwels type III fractures [3]. Pauwels’ 1935 publication was the first biomechanical classification of femoral neck fractures [4]. Although there has been some debate about the exact angles that define the categories, the underlying theoretical principle is that the more vertical the fracture line, the greater the shear force it bears, and the greater the risk of complications such as nonunion; Pauwels’ III fracture (Pauwels angle > 50°) is the most vertical [5]. Due to the large vertical shear force, the stable fixation of Pauwels III femoral neck fracture is very difficult, and the prognosis is unacceptable [6]. Arthroplasty is usually quickly ruled out for younger patients since implant normally don’t endure more than 20 years, and the surgery is associated with multiple complications including infections and aseptic loosening [7, 8]. The best fixation strategy for this fracture remains controversial [9]. The currently available internal fixation methods for femoral neck fracture include cannulated screws, dynamic hip screw (DHS), cephalomedullary nails, and proximal femoral locking plates [10–12]. However, the postoperative failure rate of these fractures is very high, ranging from 20 to 80% [13–15]. Shear force is dominant in femoral neck vertical fractures, so the internal fixator must be able to resist these shearing forces during bone healing [16]. The DHS was developed to address this issue, but their use is associated with an increased risk of osteonecrosis [17, 18]. Cannulated screws remain the most promising and commonly used devices because of their minimal invasiveness, easy handling, and ability to induce dynamic compression [19].

To improve fixation stability, Mir et al. [20] proposed the use of a medial buttress plate to treat femoral neck fractures in young adults based on previous studies. A medial buttress plate can clamp the fracture apex, neutralize shearing forces, and transfer them into compressive forces into the plane of cannulated screws or other typical construct [21]. Kunapuli and colleagues [22] first studied the strength of augmented versus nonaugmented medial buttress plates for stabilizing vertical shear femoral neck fractures and found that augmentation increased the load to failure by 183% and improved the construct’s stiffness by 35%. Li et al. [16] used finite element analysis to compare outcomes for the combination of medial buttress plate with cannulated screws to those of cannulated screws alone. The medial buttress plate combination can provide greater stability, which indicates better healing of femoral neck fractures. A recent clinical study by Ye et al. [23] reviewed the results of 28 vertical femoral neck fractures treated with cannulated screws augmented with buttress plates and found that augmentation of cannulated screw fixation resulted in an 89% union rate, which was significantly higher than the use of cannulated screws alone. As a result, conventional sliding compression fixation combined with a medial buttress plate has become the latest treatment for young patients with Pauwels III femoral neck fractures. Despite good results with this approach, there is no uniform criterion for the fixation of proximal fracture block. On one hand, a unicortical long screw may be more stable, but it will interfere with cannulated screw placement and bring great inconvenience to the operator; the single unicortical screw has little effect on cannulated screws, but it is not clear whether it offers sufficient stability is enough.

In this study, strain at different positions was measured to study the mechanical performance of two fixation methods, in addition to recording the vertical load and displacement. The bonding strain gauge (SG) was first reported in 1938, followed a few years later by the first application to bone biomechanics [24]. SGs have been widely used in bone biomechanics since; Because of their accuracy and high-frequency response, they are still the gold standard for bone strain measurement [25]. The working principle of SG is to read the increase or decrease in resistance of metal materials when they are elongated/contract by external tensile/compressive force [26].

This study tested the hypothesis that compared with a unicortical long screw, a unicortical short screw can provide the same stability. We compared the biomechanical performance of two fixation methods of cannulated screws combined with medial buttress plates on synthetic bone and assessed the biomechanical properties of these fixation methods by strain electrical measurement to provide a theoretical reference for clinical operation method selection.

## Materials and methods

### Specimen preparation

A total of 14 synthetic bone models of the right femur (Model 2200, Synbone, Zizers, Switzerland) were used for this biomechanical analyses, which allowed us to increase the sample size compared to a cadaveric study. The artificial femurs are covered with a thin layer of cortex and filled with low-density polyurethane foam corresponding to cortical and cancellous bone. The mechanical properties and cortical bone thickness are representative of cadaveric bone from younger patients [3]. The models were 455 mm in length, with a 135° neck shaft angle, 15° anteversion, 48 mm head diameter, and a minimum femoral shaft diameter of 28 mm. A protractor was used to draw the fracture line under the head of the femur (60° to the line of the horizontal). The specimen was osteotomized with a band saw, and the cross-section was perpendicular to the neck axis to simulate a Pauwels type III fracture. All samples were initially implanted with cannulated screws under fluoroscopic guidance before osteotomy to facilitate anatomical reduction. The medial buttress plate was implanted after osteotomy, because the femoral neck will be shortened. If the medial buttress plate is pre-drilled and initially implanted before osteotomy, then the broken end of the fracture will not be completely attached after reimplantation of the medial buttress plate, and anatomical reduction will not be achieved.

**Fig. 1:**
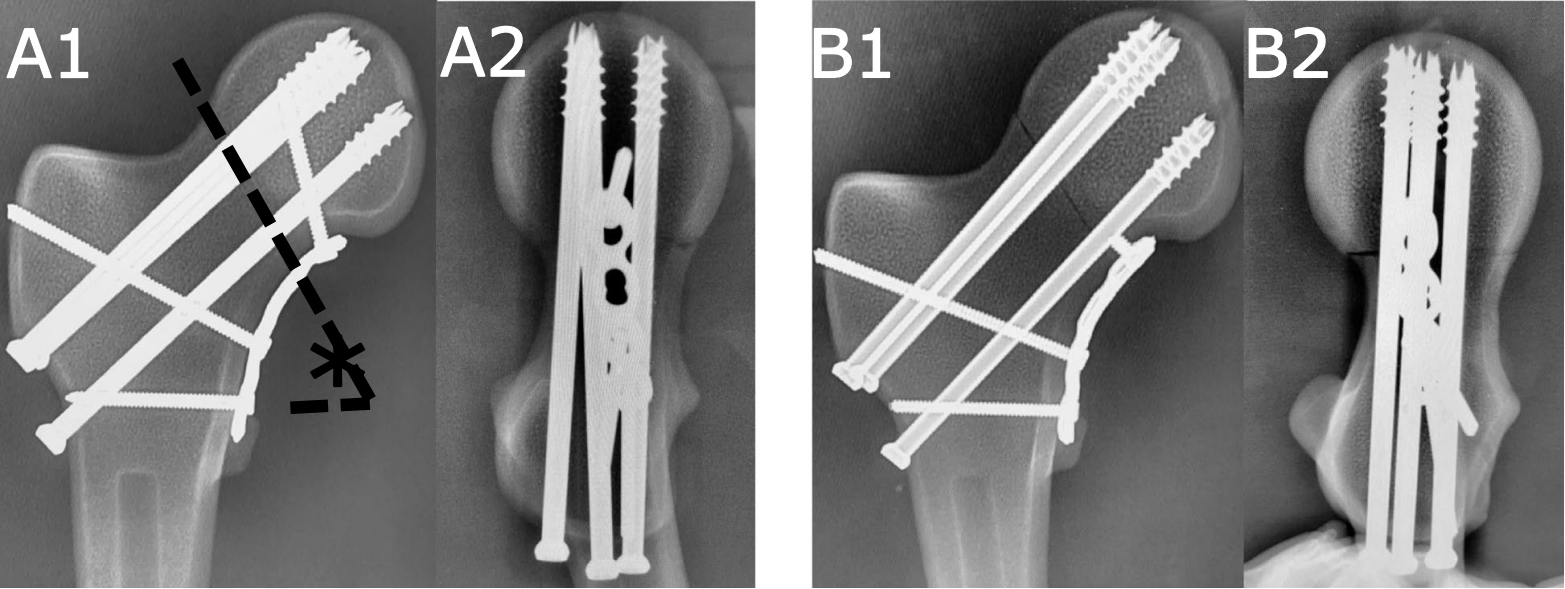
Pauwels III fractures were simulated via osteotomy by creating a fracture oriented 60° (*) from the horizonta. The two groups all used three cannulated screws combined with medial buttress plate fixation, but the fixation of the proximal fracture block was different: **A**) long screws with a length of 40 mm, **B**) Short screw with a length of 6 mm

Fourteen synthetic bone models were divided into two groups and subjected to different fixation methods (Fig. 1). All samples were fixed with three 7.0-mm cannulated screws with a triangular construction. The lowermost screw was positioned in the femoral calcar region, with the starting point above the lesser trochanter. The remaining two were located above, near the anterior or posterior cortex of the femoral neck. All screws are located 5 mm from the subchondral bone of the femoral head. A 3.5 mm thick locking plate with four holes was bent accordingly to fit the lower edge of the neck of the femur. At the distal end of the fracture line, two bicortical screws were used to fix the plate to the femur at the distal end of the fracture line. The two groups were treated as follows: Group A, the proximal end of the fracture line was fixed to the femoral head with a long unicortical screw (length 40 mm); Group B, the proximal fragment was fixed with a short unicortical screw (length 6 mm).

**Fig. 2:**
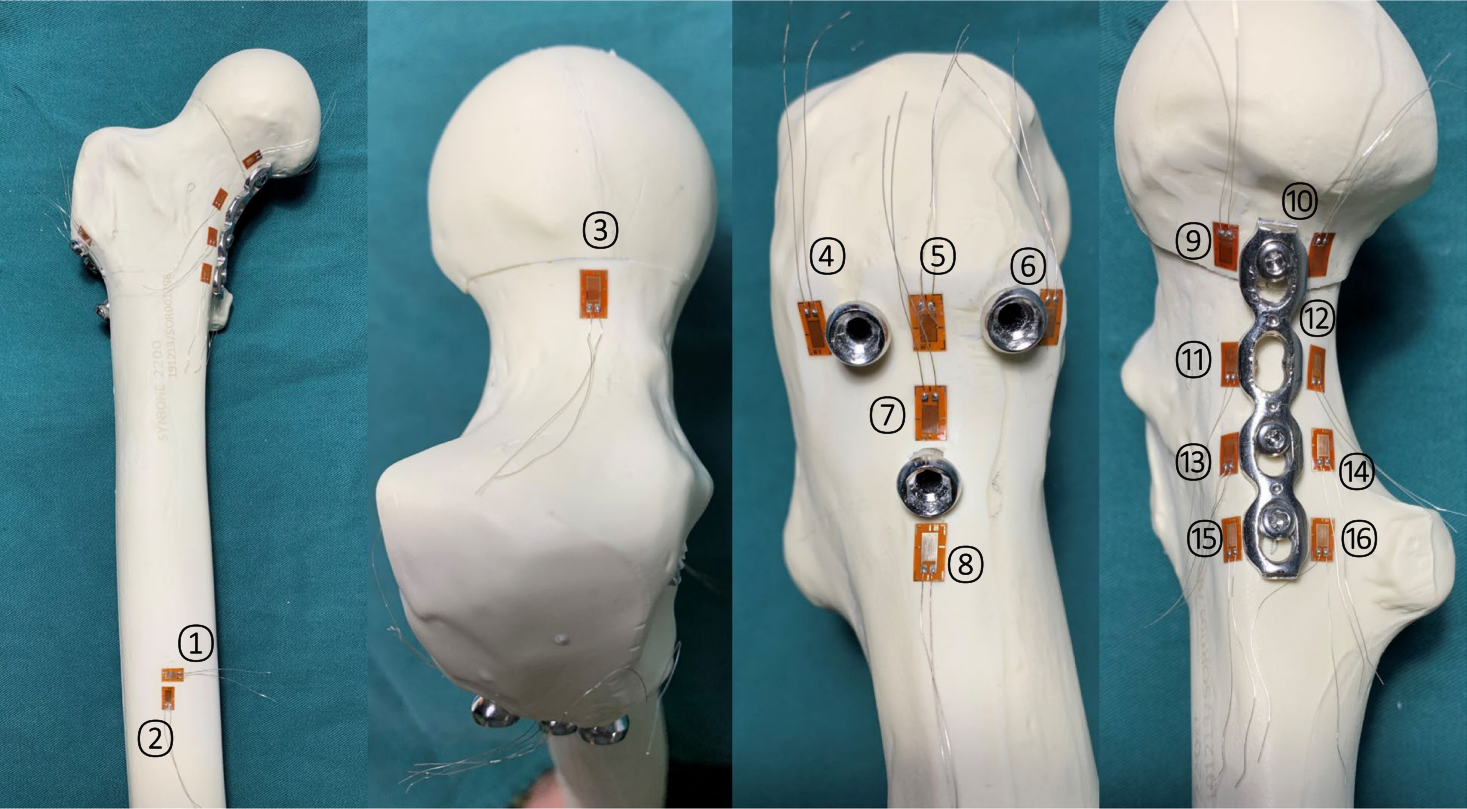
The location of the strain gauge

Synthetic femoral models were cut from the middle diaphyseal region to a final size of 200.0 mm. The distal end was firmly fixed with polymethylmethacrylate, and each specimen was oriented in 15° of adduction in the coronal plane and aligned vertically in the sagittal plane to imitate the standing posture of one leg. Adduction of 15° simulates the physiological load of the proximal femur during standing on one leg of the gait and has been used in previous biomechanical studies [27]. The bone surface was cross-smoothed with sandpaper along the SG axis at 45° [28]. The surface was cleaned with ethanol followed by acetone and allowed to dry naturally [29]. SGs (BX120-3AA, TESTING INSTRUMENT FACTORY HUANGYAN ZHEJIANG, Zhengjiang, China) were attached in the preset position of the sample with cyanoacrylate adhesive (T-1, Beihua Chemical Works, Beijing, China). The lead of the SG was welded to the adjacent terminal and connected to a strainmeter by a wire cable. The wiring method is quarter bridge, and the temperature compensation is external. The SG position distribution is shown in Fig. 2.

### Testing

**Fig. 3:**
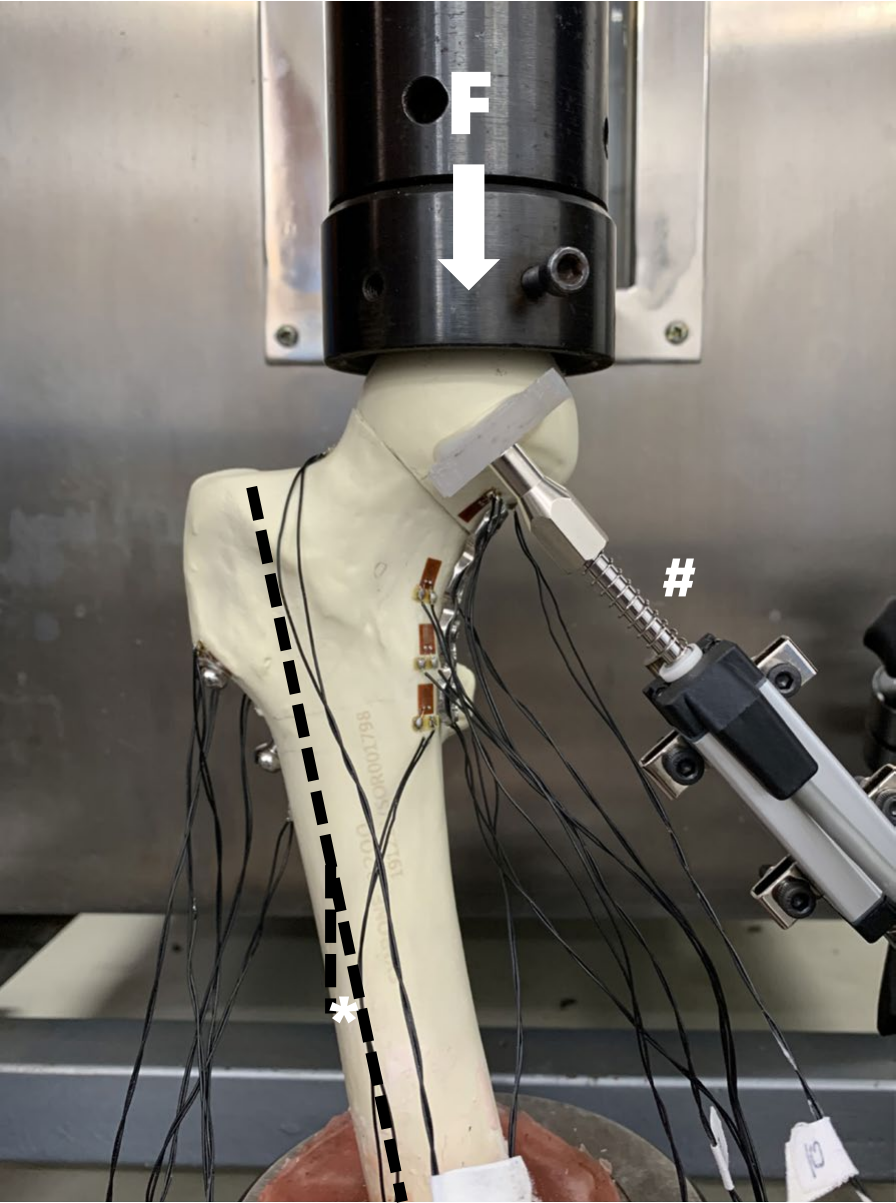
Synthetic femoral models were cut from the middle diaphyseal region with a final size of 200.0 mm. The distal end was firmly fixed with polymethylmethacrylate to each specimen was oriented in 15° (*) of of adduction in the coronal plane. The displacement transducer (#) measures the relative displacement between the fracture fragments under load

We used an MTS model 55,100 material testing machine (Material Testing Systems, MTS Systems Corp., Eden Prairie, MN, USA) with a 1000.0 KN capacity for vertical load testing. The load is transmitted through the load sensor, and the displacement is transmitted through the tie rod displacement transducer (KTR-12, MIRAN, Guangzhou, China). The displacement transducer (range 15 mm, resolution 0.01 mm) measures the relative displacement between fracture fragments under load; we chose this approach over vertical displacement because it represents the overall displacement of the model rather than the relative displacement of the fracture fragments (Fig. 3). Before starting the measurement, the specimen was preloaded to 100 N to provide close contact with the biomechanical testing machine and reduce error due to the elastic creep effect [30]. Three biomechanical experiments were carried out. 1) Strain measurement: the machine provides a vertical compression load with a peak value of 500 N at a rate of 2 mm/min and records the strain value of each measurement point at 500 N. 2) Stiffness test: a vertical compression load is provided at a device movement rate of 2 mm/min until 500 N, and use the slope of the load-displacement curve in the linear elastic region to calculate the axial stiffness. 3) Failed load test: the machine provides a vertical compression load at a rate of 2 mm/min until specimen failure. The peak load over this duration represents the structural collapse of the fixation construct and is referred to as failure load. The construct failure modes were recorded. Failure was defined as 5-mm linear or synthetic bone fracture as previously reported [10, 31, 32]. The justification for this threshold was given by Alho et al. [33], who found that a change in fracture position by 5 mm at 3-month follow-up was strongly associated with local complications and the need for revision surgery.

### Statistical analysis

All statistical calculations were carried out using SPSS (SPSS Version 26; SPSS Inc., Chicago, IL, USA). Descriptive analysis of the data was performed after confirming the normality of the data (Kolmogorov–Smirnov test). Unpaired Student’s t-test.

was used to analyze the data from the two structural groups to and identify differences in the maximum load and stiffness. Post-hoc pair-wise comparisons were made with Holm-Sidak tests. In all cases, differences were considered significant at *P* < 0.05.

## Results

We measured the strain distributions, displacements, and loads for two types of internal fixation. The values are listed in Table 1 and Table 2.

**Table 1:** Stress distribution of each group under 500 N load (Gpa)

Measuring points	Group A (SD)	Group B (SD)	P
**1**	0.29 (0.10)	0.29 (0.13)	0.945
**2**	0.66 (0.07)	0.67 (0.05)	0.793
**3**	0.70 (0.11)	0.66 (0.16)	0.606
**4**	1.26 (0.31)	1.28 (0.16)	0.906
**5**	2.00 (0.24)	1.92 (0.23)	0.535
**6**	1.24 (0.37)	1.22 (0.19)	0.915
**7**	1.41 (0.34)	1.41 (0.24)	0.972
**8**	2.80 (0.27)	2.76 (0.52)	0.874
**9**	0.63 (0.04)	0.62 (0.05)	0.721
**10**	0.6 (0.04)	0.55 (0.07)	0.136
**11**	2.12 (0.34)	2.00 (0.38)	0.541
**12**	2.20 (0.23)	2.14 (0.35)	0.722
**13**	4.83 (0.64)	4.74 (0.35)	0.764
**14**	4.57 (0.54)	4.63 (0.47)	0.835
**15**	5.79 (0.57)	5.89 (0.51)	0.716
**16**	5.88 (0.42)	6.08 (0.65)	0.502

Mean values are given along with one standard deviation in parentheses

**Table 2:** Biomechanical properties of the three fixation techniques

	Group A	Group B	P
Failure load (N)	1968.09 (227.29)	1911.86 (215.55)	0.669
Stiffness(N/mm)	399.94 (91.20)	390.70 (104.99)	0.842

Mean values are given along with one standard deviation in parentheses

### Strain measurement

We referred to the literature to calculate the stress value of each measuring point with Hooke’s law [19]: σ = E ε (where σ is stress, ε is strain, and E is the elastic modulus). The calculated elastic modulus was 1076. In terms of strain distribution, there was no significant difference between the two groups (*P* > 0.05). The stress at the joint between the medial buttress plate and screw was greater than the stress near the cannulated screws, indicating that that the plate can reduce stress. The maximum stress and strain occurred at measuring points 13, 14, 15, and 16; the addition of the medial buttress plate improved shear force resistance for fracture stability and healing. Less stress was measured near the hollow lag screw hole (5, 6, 7, and 8). The minimum stress and strain were at measuring points 3, 9 and 10 (Table 1).

### Failure load and stiffness

Among the 14 specimens, catastrophic failure occurred in 2, and the other 12 had displaced proximal fractures > 5 mm. The average maximum loads of Group A and B were 1968.09 N (SD = 227.29 N), 1911.86 N (SD = 215.55 N) respectively. There was no statistically significant difference in the average maximum load among the two groups (*P* = 0.669). The average stiffnesses of groups A and B were 390.70 N/mm (SD = 91.2 N/mm), 390.70 N/mm (SD = 104.99 N/mm) respectively (*P* = 0.842).

## Discussion

Femoral neck fractures in young adults are usually the result of high-energy trauma [34], and they are associated with high rates of postoperative osteonecrosis, nonunion, malunion, and revision surgery [13, 35, 36]. Anatomical reduction and stable internal fixation are necessary for satisfactory treatment of femoral neck fractures [37]. Some biomechanical studies reported that constructs augmented with medial buttress fixation had significantly higher stiffness and load to failure [22, 23, 38, 39]. However, the most suitable medial buttress plate fixation method is controversial. The present study evaluated and compared different kinds of internal fixation for Pauwels type III femoral neck fractures. Fourteen fracture models were generated to analyze two fixation styles under identical loading conditions. The maximum loads and strain distributions were compared to investigate biomechanical differences [38].

In terms of the fixation screws for the proximal bone block, there were no significant differences in the mechanical results when we compared the unicortical long screw and the short screw. That is, a unicortical short screw provided the same stability as a unicortical long screw.

**Fig. 4:**
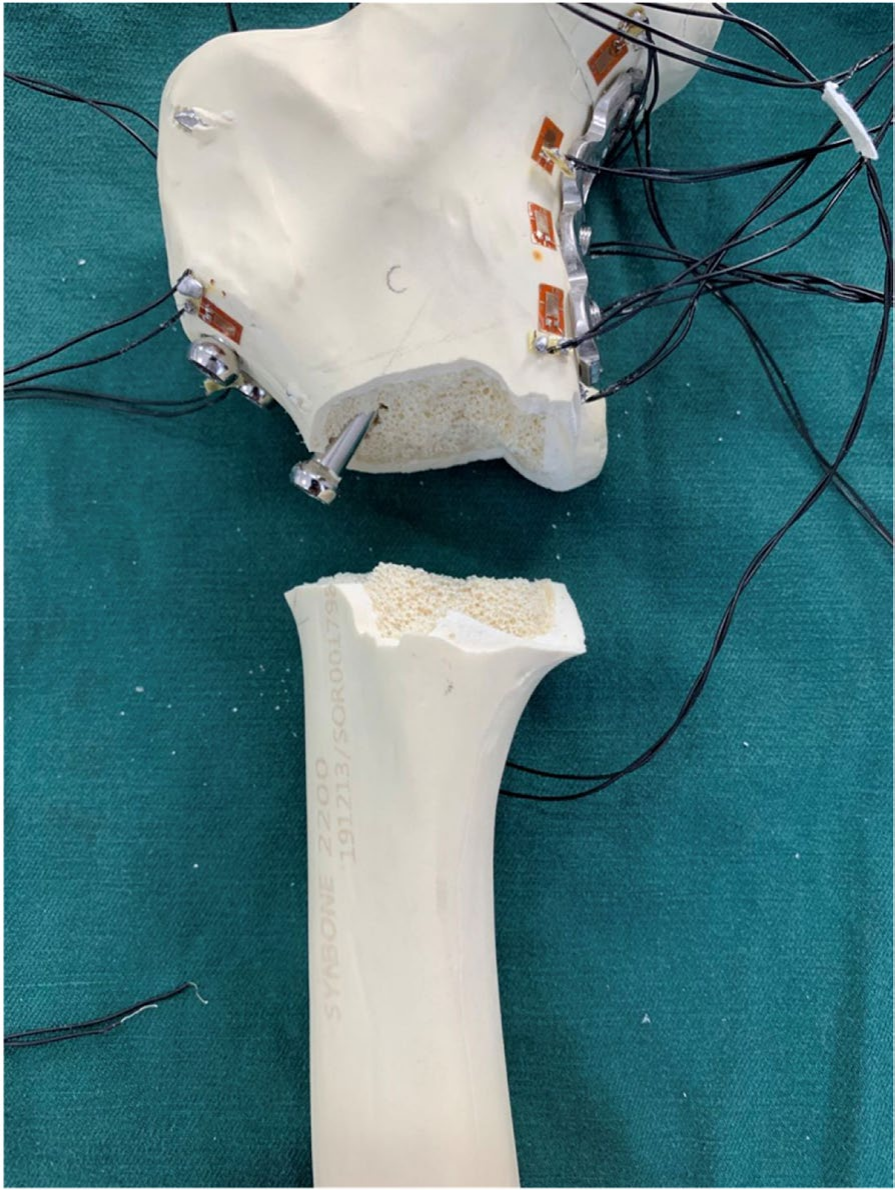
The sample fractured during the loading process. The fracture site is near the 15 and 16 measuring points, which represents the place where the stress is concentrated

The strain measurement findings revealed that the length of the screw at the proximal end of the steel plate did not obviously influence the strain distribution, which was not significantly different among the two groups. The medial buttress plate can provide an additional path to transfer force between the fractured fragments, which dissipates stress on cannulated screws and the 12 and 13 measuring points. Correspondingly, the stress increased and strain concentration occurred at the joint of the medial buttress plate and screws (13, 14, 15, and 16 measuring points; Table 1). Catastrophic failure occurred in 2 samples, and the location of the fracture corresponded to the stress concentration location (Fig. 4). Similarly, Zeng et al. performed finite element analysis and showed that strain concentration occurred at the joint between the medial buttress plate and screws [38]. However, strain is the major determinant of crack initiation and development before bone fracture [40], suggesting that stress concentrated at the junction of the plate and screw may cause periprosthetic fractures.

In this present study, we also found no difference in the maximum loads and stiffnesses of Groups A and B. In other words, a proximal fracture can be fixed with a short screw to achieve the same stability as with a long screw. The reason may be that the cortical bone has typical mechanical load-bearing characteristics; screw penetration through the cortical bone is the key, whereas screw length does not dramatically affect stability [41].

Moreover, the fixation of proximal femoral neck fractured fragments with unicortical short screw has many advantages compared with unicortical long screw. First, the unicortical short screw will not interfere with cannulated screw placement, making the operation more convenient for the surgeon. Secondly, the unicortical short screw will not enter the hip joint cavity, prevents the screw from being too long to damage the articular surface. Finally, the unicortical short screw minimizes the damage to the blood supply of the femoral head.

There are some limitations of the current study. First, we employed synthetic bones rather than cadaveric bones, so the results do not directly translate to the anatomy of the femoral trabeculae and the forces they can withstand. The artificial setting also does not truly reproduce the manner in which this fracture develops. Synthetic bones also eliminate patient variables and ensured that the biomechanical properties were comparable between groups. Second, femoral neck fractures were idealized by making smooth saw cuts perpendicular to the neck axis. This may not accurately simulate the jagged features of the interface between bone fragments in clinical situations. Specifically, the lower interface roughness may have underestimated stiffness and strength values. Thirdly, we lengthened the screw channel at the proximal end of the buttress plate by 2 mm, but it remains unclear how much enlargement is optimal.

## Conclusions

Our study found no statistically significant difference in axial load models between unicortical short locking screws (or combined screw channel enlargement) and unicortical long locking screws. The application of a unicortical short screw fixes the proximal end of the medial buttress plate and expands the screw path to the proximal end, which might be a viable surgical plan.

## Data Availability

All data generated or analyzed during this study are included in this article.
